# Data-intelligence-driven flood forecasting and early warning in a small river basin: A BiLSTM model integrated with the “631” response mechanism

**DOI:** 10.1371/journal.pone.0357040

**Published:** 2026-08-28

**Authors:** Peisheng Yang, Xiaohua Xu, Meilan Shao, Yewei Liu, Longhui Zhu

**Affiliations:** 1 Jiangxi Academy of Water Science and Engineering, Nanchang, China; 2 Jiangxi Key Laboratory of Flood and Drought Disaster Defense, Nanchang, China; 3 Jiangxi College of Construction, Nanchang, China; Leibniz University Hannover, GERMANY

## Abstract

Reliable flood forecasts must be translated into timely, accountable warnings, particularly in small mountainous basins with short concentration times and limited historical records. This study links a Bidirectional Long Short-Term Memory (BiLSTM) rainfall–runoff model with Jiangxi Province’s hierarchical “631” response mechanism. Twenty flood events from the Xiangshui River Basin were divided chronologically into 14 training events and six independent test events. The final network contains four stacked BiLSTM layers and a fully connected output layer; it does not contain convolutional or pooling layers. Against LSTM, GRU and Xin’anjiang baselines, BiLSTM obtained a mean Nash–Sutcliffe efficiency of 0.90 and a mean denormalised RMSE of 11.2 m^3^/s. All six paired NSE differences favoured BiLSTM, but the small test set precludes confirmatory statistical inference; results are therefore reported with event-level differences and leave-one-event-out sensitivity rather than interpreted from the Wilcoxon p-value alone. SHAP marginal attribution identified Qingxi, Yingfang and Zhongcun as the most influential stations and the two most recent input lags as the dominant time steps. A marginal-consistent station–lag diagnostic further localised the strongest first-order contributions to upstream rainfall at t−1 and t−2, while explicitly not being treated as a SHAP interaction estimate. Forecast skill declined from NSE = 0.90 at 1 h to 0.58 at 6 h, indicating that quantitative precipitation forecasts are required for operational 3–6 h prediction. The revised integration framework therefore assigns observation-driven BiLSTM forecasts to the short-lead “1” stage and QPF-informed forecasts to the “3” and “6” stages. The findings support a focused, auditable pathway from data-driven prediction to warning action while defining the limits imposed by event scarcity and missing future rainfall forcing.

## Introduction

Flood disasters rank among the most frequent and destructive natural hazards worldwide, and their severity is increasing under climate change and rapid urbanisation [1,2]. In China, small and medium river basins (<3,000 km^2^) account for over 70% of flood-related fatalities due to rapid hydrological response, sparse monitoring and complex underlying surfaces [3,4]. Enhancing both forecast *accuracy* and warning *effectiveness* is therefore a pressing need [5].

Traditional physically based models—Sacramento [6], HBV [7] and Xin’anjiang [8]—require extensive calibration and often underperform in data-scarce catchments [9,10]. Data-driven methods have emerged as powerful alternatives. Machine learning models including support vector machines and random forests demonstrate strong nonlinear fitting [11,12], while deep learning—particularly LSTM networks—has achieved state-of-the-art performance [13]. BiLSTM extends LSTM by simultaneously learning forward and backward temporal dependencies, beneficial for capturing asymmetric flood hydrographs [14–16].

Several gaps remain. First, most BiLSTM applications target large, well-gauged basins, whereas small mountainous catchments provide fewer events and shorter warning windows [17,18]. Second, recent two-dimensional hidden-layer, attention and Pathformer architectures can represent richer spatio-temporal dependence [19–21], but their data requirements and multi-step forcing assumptions differ from those of event-limited operational basins. Third, hydrological explainability studies increasingly combine predictive models with SHAP, yet many report only global feature rankings and do not distinguish marginal attribution from feature interaction [22,23]. Fourth, significance claims are often made from very small event sets without reporting the discreteness and uncertainty of non-parametric tests. Finally, forecasting and downstream warning actions are usually evaluated separately, leaving the operational use of model outputs insufficiently specified.

On the warning side, China has developed multiple “call-and-response” mechanisms. Zhejiang uses digital-twin acceleration [24]; Hunan has established a five-element risk-control map [25]; Gansu has deployed technology-monitoring plus grid-management [26]. Jiangxi’s “631” mechanism [27,28]—6-hour provincial, 3-hour municipal and 1-hour county-level warnings—has shown high effectiveness but lacks rigorous evaluation, cross-provincial comparison and explicit model integration.

This study makes four contributions: (i) it provides a reproducible description of a four-layer BiLSTM model and evaluates it against three deliberately low- to moderate-complexity baselines under a common event split; (ii) it reports event-level paired differences and leave-one-event-out sensitivity, avoiding confirmatory claims from six test events; (iii) it augments station- and lag-level SHAP marginals with a transparent station–lag decomposition that identifies first-order spatio-temporal concentration without mislabelling it as interaction SHAP; and (iv) it maps the forecast information available at 1, 3 and 6 h to distinct stages of the “631” warning mechanism.

### Related work

Flood forecasting has evolved from conceptual rainfall–runoff models to recurrent, attention-based and hybrid data–physics systems [29]. LSTM and GRU remain widely used because they can be trained with moderate sample sizes, while BiLSTM processes a fixed antecedent window in both chronological directions [13,14]. Transformer and Informer models improve long-range dependency modelling [30,31]; two-dimensional hidden-layer networks explicitly encode flood spatio-temporal structure [19]; DTTR combines deep temporal attention, convolution and multimodal fusion [20]; and Pathformer dynamically represents multiple temporal scales for multi-step flood forecasting [21]. Recent limited-data and hybrid studies further show the value of hydrological constraints or model ensembles [18,32]. Explainable hydrological forecasting has also expanded from global importance plots toward local and uncertainty-aware SHAP analysis [23].

These method classes are not directly interchangeable. Transformer-family and multimodal models are most informative when long records, future meteorological forcing or dense multi-basin samples are available, whereas the present archive contains only 20 discrete flood events and no QPF inputs. We therefore do not claim state-of-the-art superiority over these architectures and do not compare published NSE values across different basins. Table 1 states the experimental and literature-comparison scope explicitly.

**Table 1:** Scope of the forecasting-method comparison.

Method class	Main strength	Typical additional requirement	Role in this study
Xin’anjiang	Process-based rainfall–runoff representation	Calibrated basin parameters	Experimental baseline
LSTM / GRU	Data-efficient recurrent sequence learning	Antecedent observations	Experimental baselines
BiLSTM	Bidirectional encoding of the observed input window	Antecedent observations	Proposed experimental model
Transformer / Informer / Pathformer	Long-range and multi-scale attention	Larger samples; often multi-step forcings	Literature context; future benchmark
2-D hidden / DTTR / multimodal models	Explicit spatial–temporal or multimodal fusion	Richer spatial covariates and longer records	Literature context
Physics-informed / hybrid models	Hydrological constraints and transferability	Process variables or differentiable components	Literature context; future direction

Flash-flood warning systems include rainfall-threshold-based approaches (US FFG [33]) and simulation-based systems (EFAS [34]). The “last-mile” problem—ensuring warnings reach end-users—remains a global challenge [35,36]. Table 2 compares four Chinese provincial mechanisms.

**Table 2:** Qualitative comparison of selected provincial early warning mechanisms in China.

Feature	Jiangxi “631”	Zhejiang	Hunan	Gansu
Trigger logic	6/3/1-h staged response	Dynamic digital platform	Multi-element risk map	Monitoring plus grid response
Delivery / action	Calls, messaging and local action	Platform-based dissemination	Map-guided local control	Grid-based communication
Feedback feature	Call-back and field confirmation	Platform records	Map-based reporting	Grid reporting
Distinctive strength	Time-specific accountability chain	Rapid digital integration	Spatial risk context	Coverage through local grids

## Materials and methods

### BiLSTM architecture

#### LSTM fundamentals.

LSTM addresses the vanishing-gradient problem through gating mechanisms. At time step *t*, given hidden state $h_{t-1}$ and input $x_{t}$, the forget gate, input gate and candidate memory, cell-state update and output gate are defined as follows:


$$\begin{array}{c} \begin{array}{ccc} f_{t} & = & \sigma (W_{f}\cdot [h_{t-1},x_{t}]+b_{f}), \end{array} \end{array}$$ (1)

$$\begin{array}{c} \begin{array}{ccc} i_{t} & = & \sigma (W_{i}\cdot [h_{t-1},x_{t}]+b_{i}), \end{array} \end{array}$$ (2)

$$\begin{array}{c} \begin{array}{ccc} {\tilde{C}}_{t} & = & \tanh(W_{C}\cdot [h_{t-1},x_{t}]+b_{C}), \end{array} \end{array}$$ (3)

$$\begin{array}{c} \begin{array}{ccc} C_{t} & = & f_{t}⊙C_{t-1}+i_{t}⊙{\tilde{C}}_{t}, \end{array} \end{array}$$ (4)

$$\begin{array}{c} \begin{array}{ccc} o_{t} & = & \sigma (W_{o}\cdot [h_{t-1},x_{t}]+b_{o}), \end{array} \end{array}$$ (5)

$$\begin{array}{c} \begin{array}{ccc} h_{t} & = & o_{t}⊙\tanh(C_{t}), \end{array} \end{array}$$ (6)

where σ(·) is the sigmoid function, ⊙ denotes element-wise multiplication, *W* are weight matrices and *b* are bias terms.

#### Bidirectional architecture.

BiLSTM processes sequences in both directions. The forward (${\vec{h}}_{t}$) and backward (${\overset{\leftarrow }{h}}_{t}$) hidden states are concatenated:


$$\begin{array}{c} h_{t}=[{\vec{h}}_{t};{\overset{\leftarrow }{h}}_{t}]. \end{array}$$ (7)

**Fig 1:**
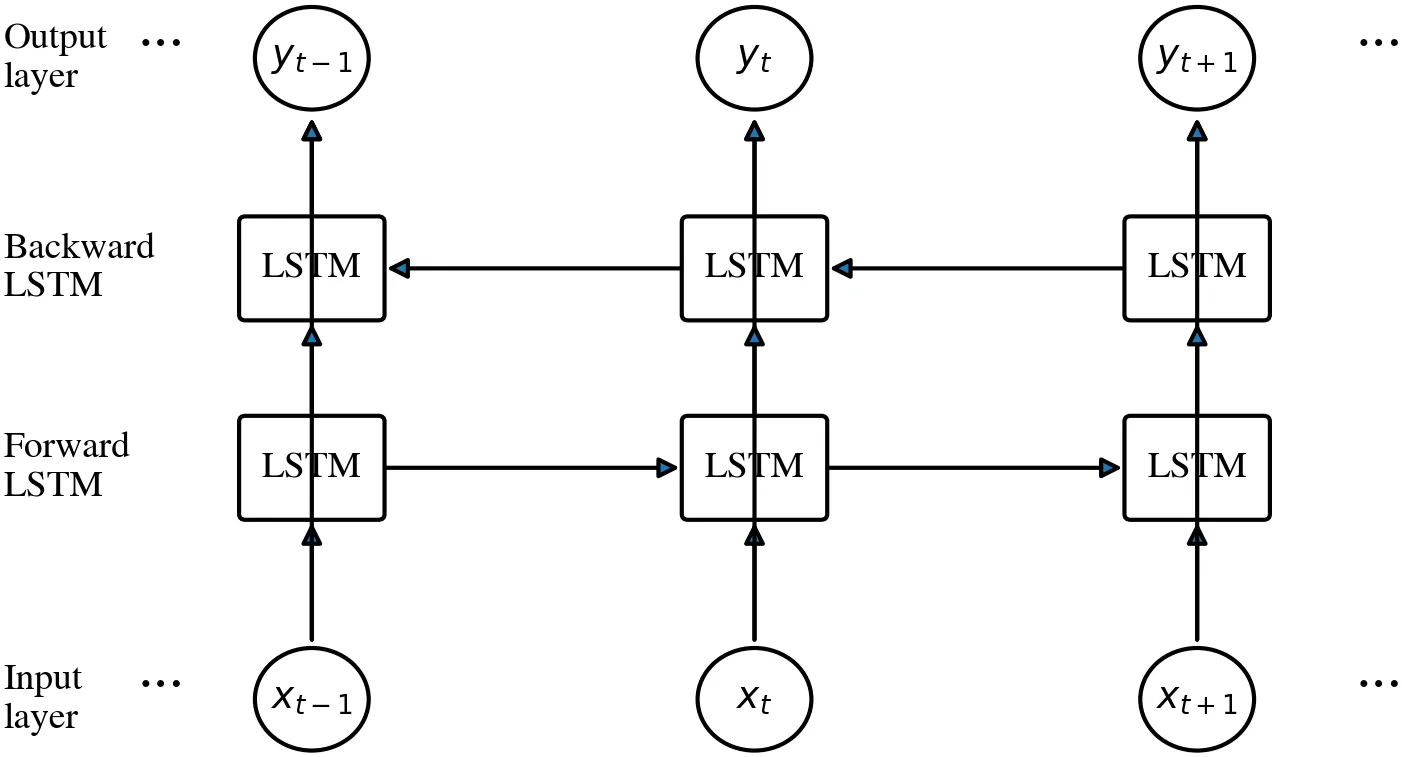
BiLSTM bidirectional architecture. Forward LSTM processes $x_{1}\to x_{5}$; backward LSTM processes $x_{5}\to x_{1}$. Hidden states are concatenated at each step.

Both directions operate only on the five observed antecedent steps: the forward layer reads t−5→t−1 and the backward layer reads the same window in reverse. No value at or after the prediction target *t* is supplied, so bidirectional encoding does not introduce fu*t*ure-information leakage. Fig 1 shows the architecture.

### Study area and data processing

#### Study area.

**Fig 2:**
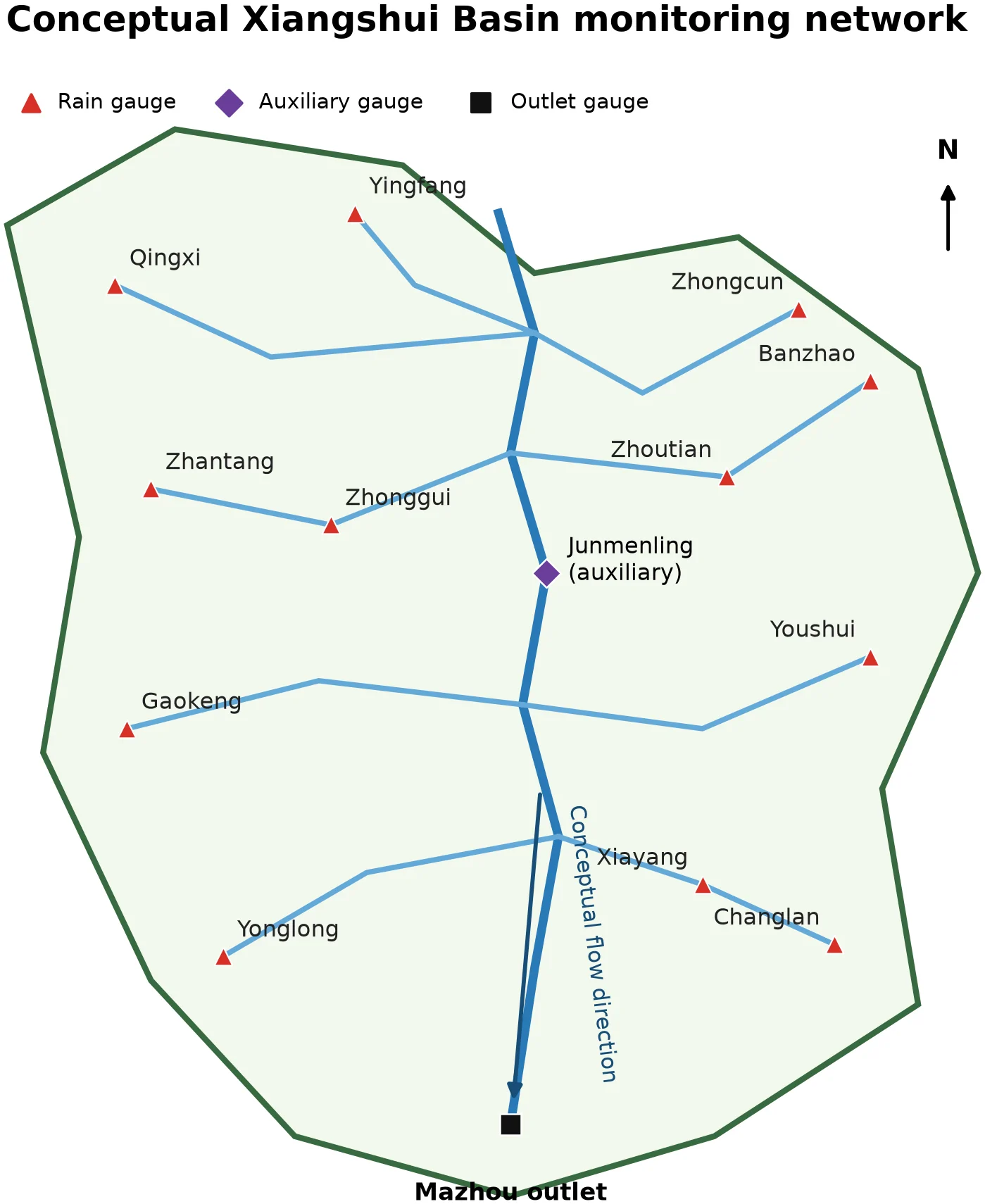
Overview of the Xiangshui River Basin. Twelve model-input rain gauges, the Mazhou outlet station and the auxiliary Junmenling hydrological station are shown. Junmenling is included for monitoring context but is not among the 12 rainfall input features. This conceptual monitoring-network diagram was drawn specifically for this article, is not to scale and uses no external basemap, satellite image or third-party geographic layer.

The Xiangshui River is a primary tributary of the Gongjiang River in the upper Ganjiang system, Jiangxi Province. The full basin covers 2,056 km^2^; the Mazhou outlet controls the 1,758 km^2^ modelling domain used in this study. River-network density is 0.217 km/km^2^. The subtropical monsoon climate concentrates more than half of annual rainfall in April–July. Available basin records describe a predominantly hilly and mountainous catchment, with short steep hillslopes, narrow valleys, shallow weathered soils in headwater areas, forest-dominated uplands, cultivated valley floors and dispersed rural settlements. Flood generation is therefore primarily storm-driven: intense convective or frontal rainfall produces rapid infiltration-excess and saturation-excess runoff, followed by short channel concentration to Mazhou. These characteristics provide a physical basis for the strong attribution of recent upstream rainfall, while also limiting transferability to flat, snowmelt-dominated or highly urbanised basins. Twelve automatic rain gauges are distributed throughout the modelling domain (Fig 2).

#### Data processing.

**Normalisation.** Min–max normalisation compresses variables to [0,1]:


$$\begin{array}{c} x^{\prime }=\frac{x-x_{\min}}{x_{\max}-x_{\min}}. \end{array}$$ (8)

Denormalisation follows $\hat{Q}=x^{\prime }(Q_{\max}-Q_{\min})+Q_{\min}$, where $Q_{\max}=1180$ m^3^/s and $Q_{\min}=12$ m^3^/s. All errors are reported in both normalised and physical units.

**Dataset partitioning.** Twenty flood events were split temporally into 14 training (2012–2022) and 6 independent test (2023–2024) events (Table 3). Rows were not randomly reassigned across events.

**Table 3:** Flood event dataset division.

No.	Event	Set	No.	Event	Set
1	2012-08-04	Train	11	2020-06-08	Train
2	2013-06-05	Train	12	2021-05-17	Train
3	2014-05-23	Train	13	2021-06-05	Train
4	2015-05-26	Train	14	2022-06-08	Train
5	2016-03-23	Train	15	2023-03-26	Test
6	2016-04-10	Train	16	2023-05-07	Test
7	2016-04-27	Train	17	2024-04-06	Test
8	2017-06-20	Train	18	2024-04-26	Test
9	2019-06-11	Train	19	2024-05-22	Test
10	2019-06-23	Train	20	2024-06-16	Test

**Fig 3:**
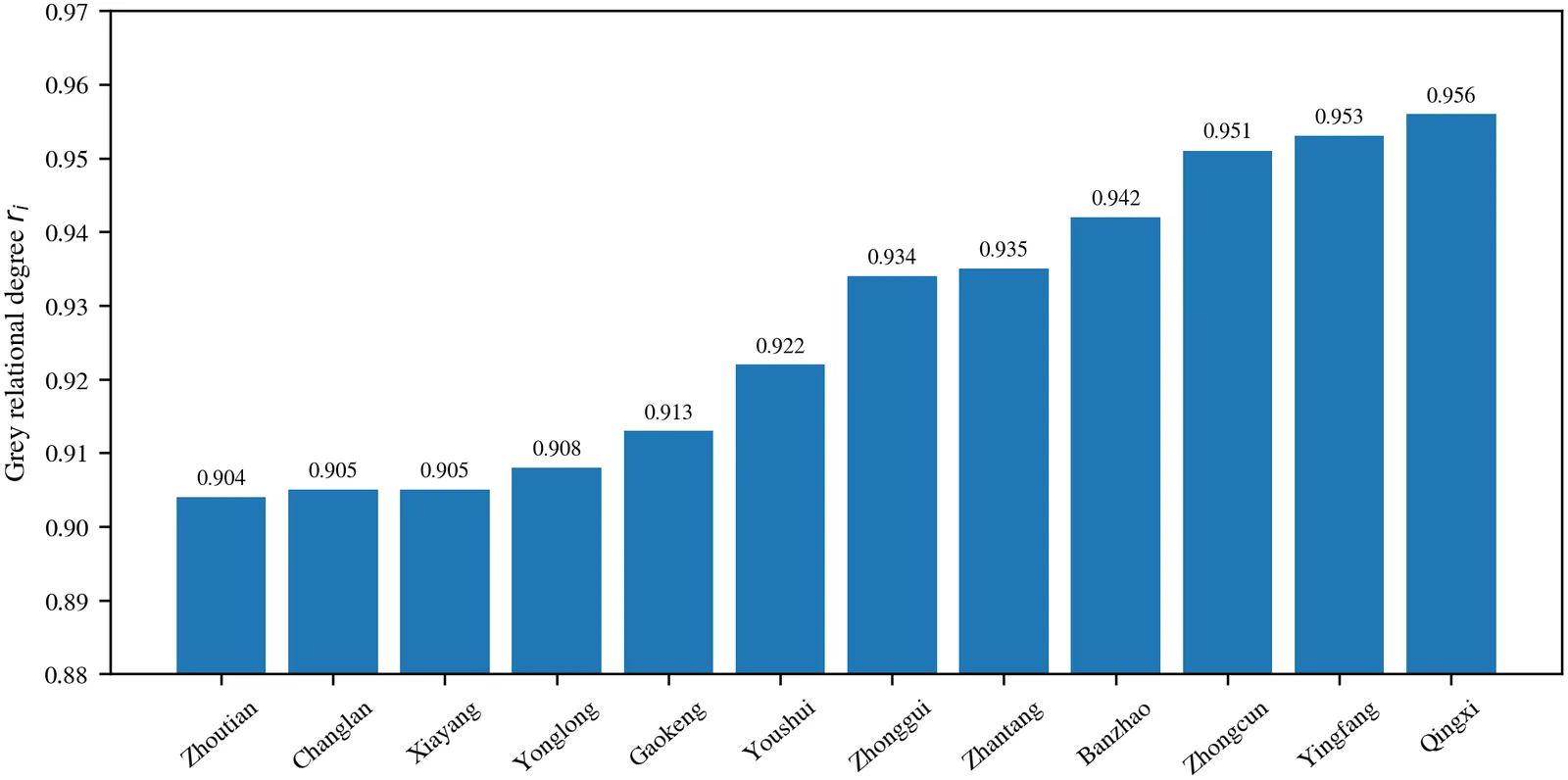
Grey relational degree of each rain-gauge station with outlet flow (ascending order).

**Grey relational analysis (GRA).** Feature relevance was assessed using GRA with a 5-hour window:


$$\begin{array}{c} \xi_{i}(k)=\frac{\min_{i}\min_{k}|x_{0}(k)-x_{i}(k)|+\rho \max_{i}\max_{k}|x_{0}(k)-x_{i}(k)|}{\left|x_{0}(k)-x_{i}(k)|+\rho \max_{i}\max_{k}|x_{0}(k)-x_{i}(k)\right|}, \end{array}$$ (9)

with ρ=0.5. The grey relational degree $r_{i}=\frac{1}{n}\sum_{k=1}^{n}\xi_{i}(k)$. All 12 stations show $r_{i}>0.9$ (Table 4), confirming high relevance; Fig 3 visualises the station ranking.

**Table 4:** Grey relational degree between station rainfall and Mazhou flow.

Station	$r_{i}$	Station	$r_{i}$
Gaokeng	0.913	Zhonggui	0.934
Changlan	0.905	Zhoutian	0.904
Youshui	0.922	Banzhao	0.942
Yonglong	0.908	Qingxi	0.956
Zhantang	0.935	Xiayang	0.905
Zhongcun	0.951	Yingfang	0.953

### Model construction and hyperparameter optimisation

**Fig 4:**
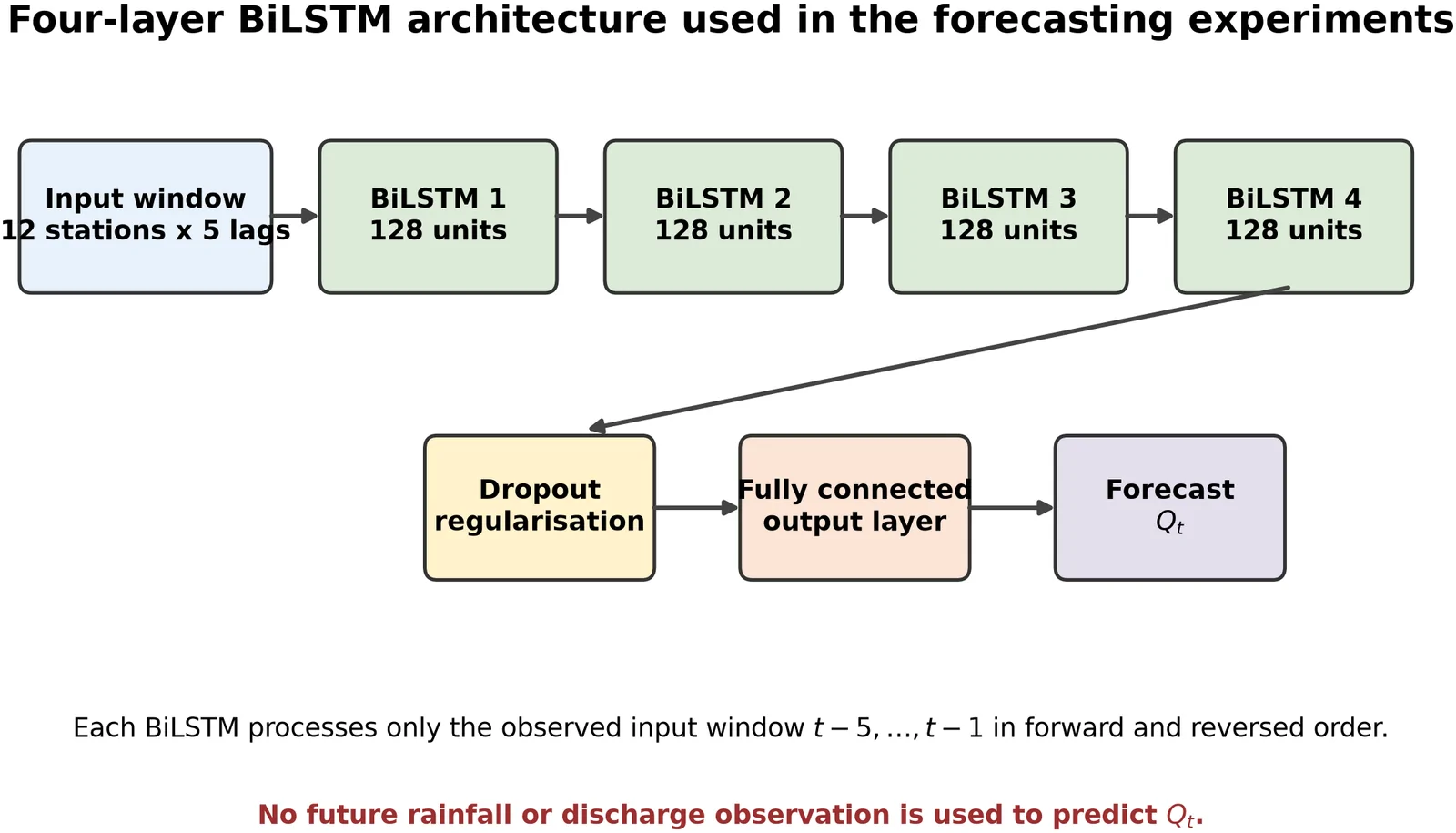
BiLSTM neural-network structure for the Xiangshui River Basin. Input: rainfall at 12 stations over lags t−5 to t−1; hidden representation: four stacked BiLSTM layers; output: one-hour-ahead flow $Q_{t}$. The backward direction reverses only the observed input window.

**Fig 5:**
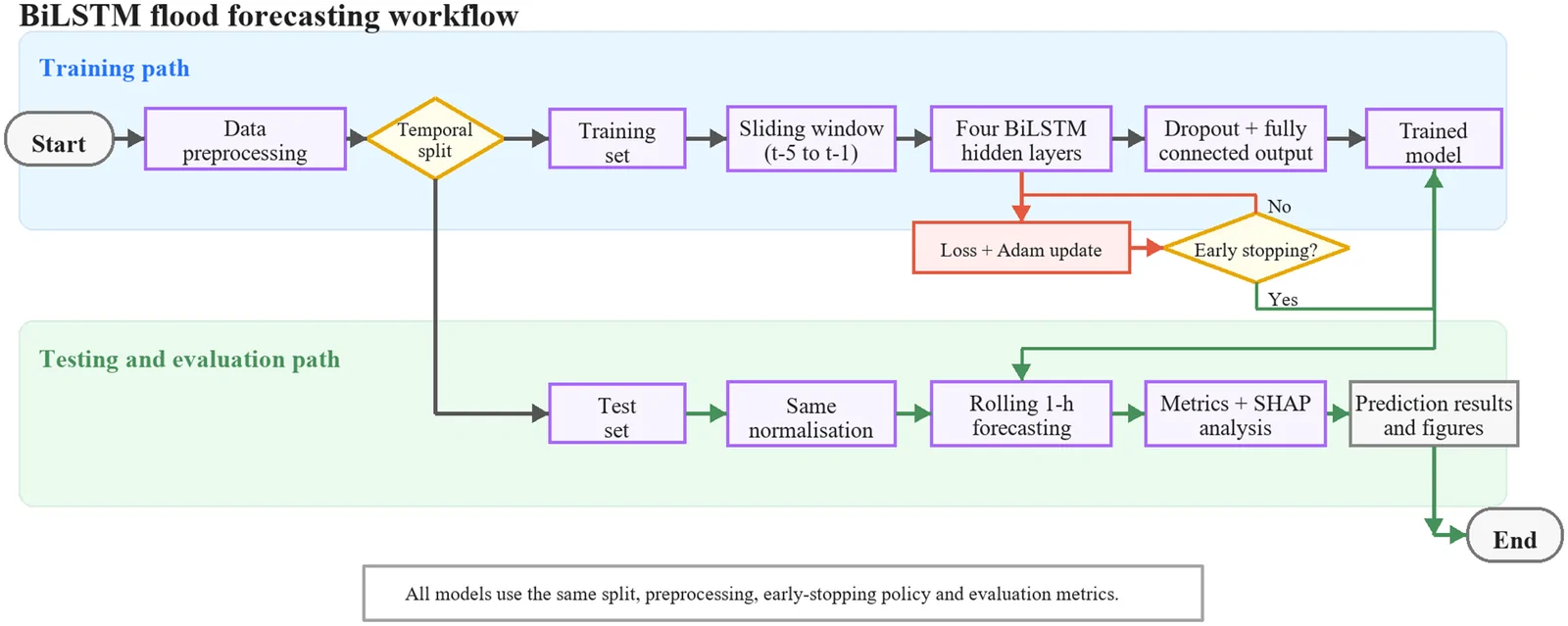
Flowchart of the BiLSTM flood forecasting methodology.

For target flow $Q_{t}$, the input tensor contains rainfall from 12 stations at lags t−5 to t−1 (12×5 features). The final network consists of four stacked BiLSTM layers with 128 units per layer, dropout regularisation between recurrent layers and a one-unit fully connected output. It contains no convolutional or max-pooling layer. The architecture in Fig 4 and the workflow in Fig 5 now use the same model name, layer sequence, input window and target notation.

Hyperparameters were tuned via grid search with early stopping (patience = 15 epochs). The optimal combination was 128 units, 150 epochs and batch size 128 (Table 5).

**Table 5:** Model performance under representative hyperparameter combinations.

Units	Epoch	Batch	MSE	Peak flow qual.	NSE	RMSE
32	25	32	0.028	66.7%	0.85	0.014
64	80	64	0.022	83.3%	0.89	0.013
128	100	64	0.020	83.3%	0.89	0.010
**128**	**150**	**128**	**0.018**	**100%**	**0.92**	**0.009**
256	180	128	0.020	83.3%	0.89	0.011

**Fig 6:**
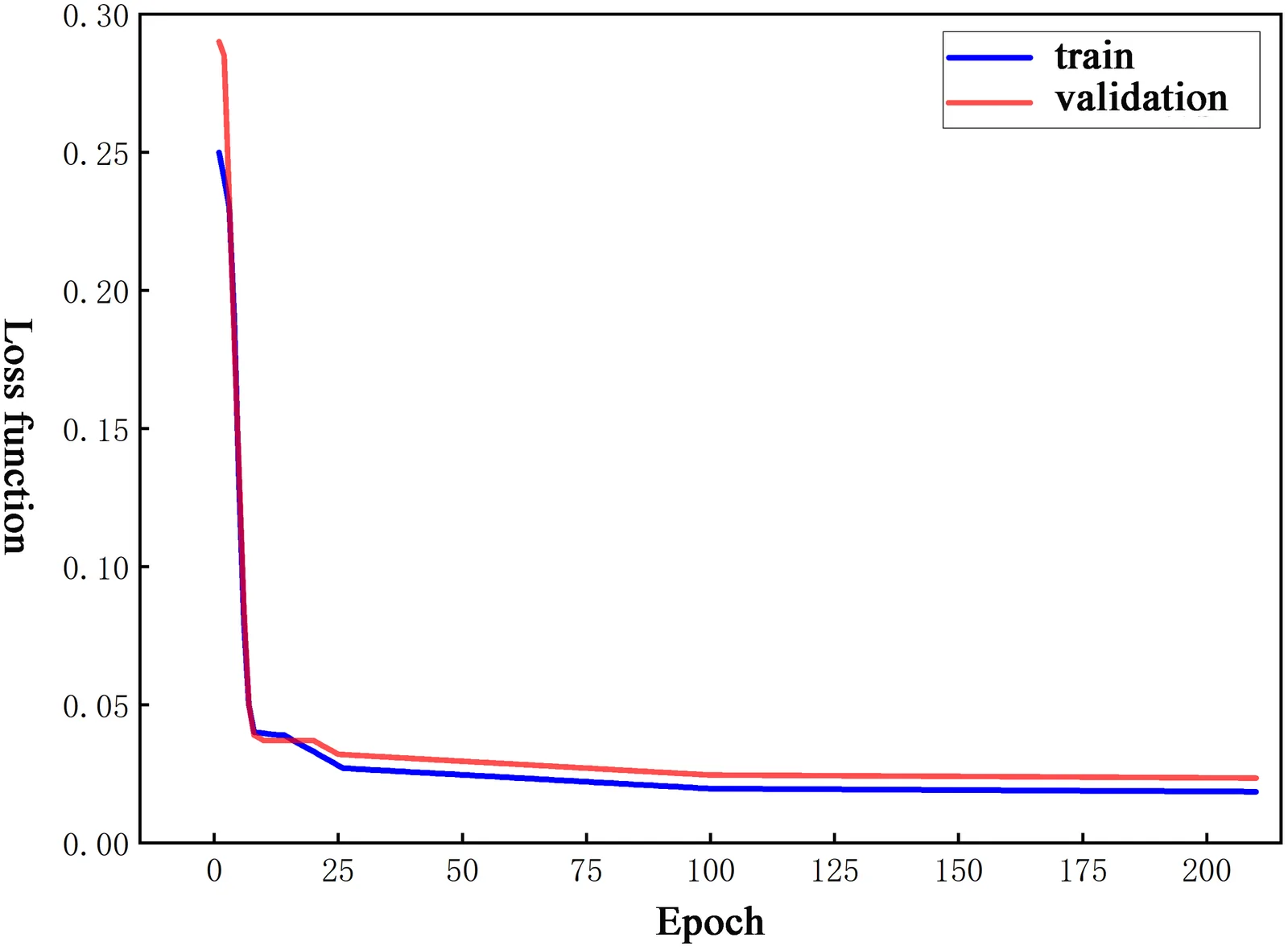
Training and validation loss curves (128 units, batch 128). Validation loss stabilises after epoch 120.

The training curve (Fig 6) confirms convergence by epoch 120 without overfitting.

### Performance metrics

The *peak flow qualification rate* is defined as the fraction of events with |peak error|≤20%. The *peak timing qualification rate* is the fraction with $|\Delta t_{p}|\leq 2$ h. The Nash–Sutcliffe efficiency and root mean square error are


$$\begin{array}{c} \text{NSE}=1-\frac{\sum_{i=1}^{n}(Q_{\text{obs},i}-Q_{\text{sim},i}{)}^{2}}{\sum_{i=1}^{n}(Q_{\text{obs},i}-{\bar{Q}}_{\text{obs}}{)}^{2}}, \end{array}$$ (10)

$$\begin{array}{c} \text{RMSE}=\sqrt{\frac{1}{n}\sum_{i=1}^{n}(Q_{\text{obs},i}-Q_{\text{sim},i}{)}^{2}}. \end{array}$$ (11)

### Baseline models and comparison scope

Three baselines were trained on identical data: (i) **LSTM** with the same recurrent depth but no backward pass; (ii) **GRU** with four layers and 128 units; and (iii) the **Xin’anjiang** conceptual model calibrated with SCE-UA [8]. All deep-learning models used the same chronological split, normalisation and early-stopping policy. The software supplement documents deterministic seed handling and dependency versions for the released reimplementation; the original analysis environment was not recoverable from the archived materials.

Transformer, Informer, two-dimensional hidden-layer, DTTR, Pathformer and hybrid physics–learning methods are discussed in Table 1 but are not assigned numerical results in this dataset. Training such high-capacity models on 14 event sequences without their intended future forcings would produce a poorly identified comparison, while importing published scores from other basins would be invalid. Consequently, the quantitative claim is restricted to the four models actually evaluated, and advanced-model benchmarking is identified as a required next step once multi-basin records and QPF inputs are available.

### SHAP interpretability

KernelSHAP [22] was used to estimate mean absolute contribution values for the 60 station–lag input features. The supplied analysis files contain two verified marginal summaries: station means $S_{s}$ and lag means $T_{l}$. To avoid presenting an unverified interaction matrix, we derive a transparent marginal-consistent first-order diagnostic,


$$\begin{array}{c} I_{s,l}=S_{s}\frac{T_{l}}{\sum_{l}T_{l}}, \end{array}$$ (12)

which distributes each station’s marginal importance across lags according to the observed temporal marginal. $I_{s,l}$ preserves the station ranking and temporal concentration but assumes separability; it is therefore labelled an attribution index rather than a SHAP interaction value. Genuine interaction SHAP requires the original sample-level 60-feature attribution matrix, which was not available in the archived analysis; the present station–lag surface is therefore interpreted only as a separable first-order diagnostic.

### Small-sample paired analysis

The six independent test events are too few for a well-powered confirmatory comparison. We therefore treat the Wilcoxon signed-rank result as exploratory and place emphasis on the paired NSE differences themselves. For each baseline we report the number of positive pairs, median and range of ΔNSE, the exact one-sided sign-test probability for six positive differences (1/2^6^ = 0.0156), and the range of the mean difference under leave-one-event-out deletion. The sign-test probability describes directional consistency under a sharp null; it does not provide a precise population effect size. No multiple-comparison-adjusted claim of superiority is made.

## Results

### BiLSTM simulation results

Table 6 presents the test-period results for six flood events. BiLSTM achieves 100% qualification rates for both peak flow and peak timing, a mean absolute peak error of 6.98%, a mean NSE of 0.90 and a mean denormalised RMSE of 11.2 m^3^/s.

**Table 6:** BiLSTM test-period results with denormalised error metrics.

Event	*Q*_obs_	*Q*_sim_	Peak err.	$\Delta t_{p}$	NSE	RMSE	RMSE	Qual.
	(m^3^/s)	(m^3^/s)	(%)	(h)		(norm.)	(m^3^/s)	
2023-03-26	758	798	+5.28	0	0.89	0.012	14.0	Yes
2023-05-07	962	921	−4.26	−1	0.92	0.010	11.7	Yes
2024-04-06	351	390	+11.11	−2	0.74	0.018	21.0	Yes
2024-04-26	1010	1098	+8.71	+1	0.94	0.008	9.3	Yes
2024-05-22	404	430	+6.44	−1	0.96	0.007	8.2	Yes
2024-06-16	984	1044	+6.10	+1	0.96	0.006	3.0	Yes
**Mean**	—	—	**6.98**	**1.0**	**0.90**	**0.010**	**11.2**	**100%**

**Fig 7:**
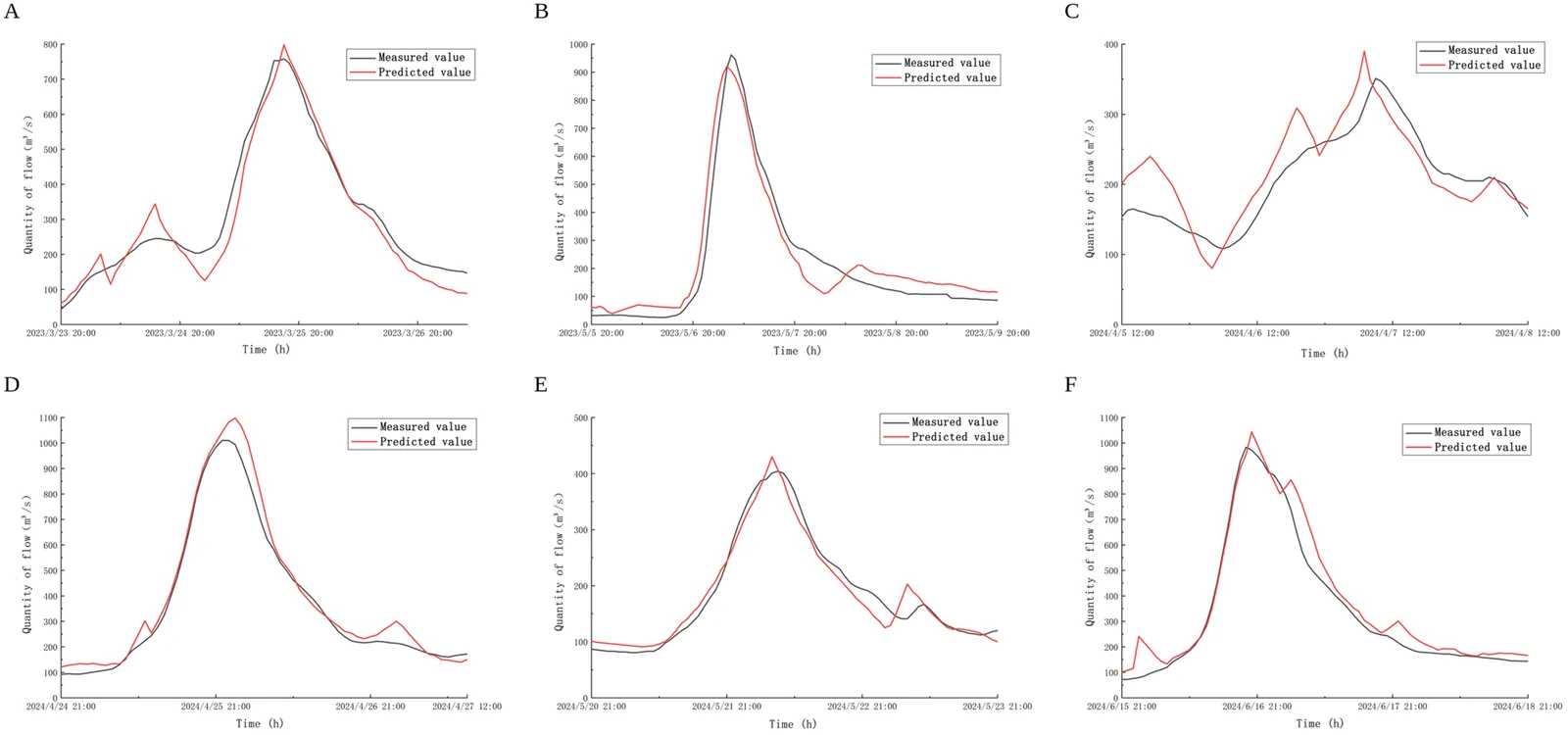
Observed (black) and BiLSTM-predicted (red) flood hydrographs for six test events. A: 2023-03-26. B: 2023-05-07. C: 2024-04-06. D: 2024-04-26. E: 2024-05-22. F: 2024-06-16.

Fig 7 shows observed versus predicted flood hydrographs.

**Fig 8:**
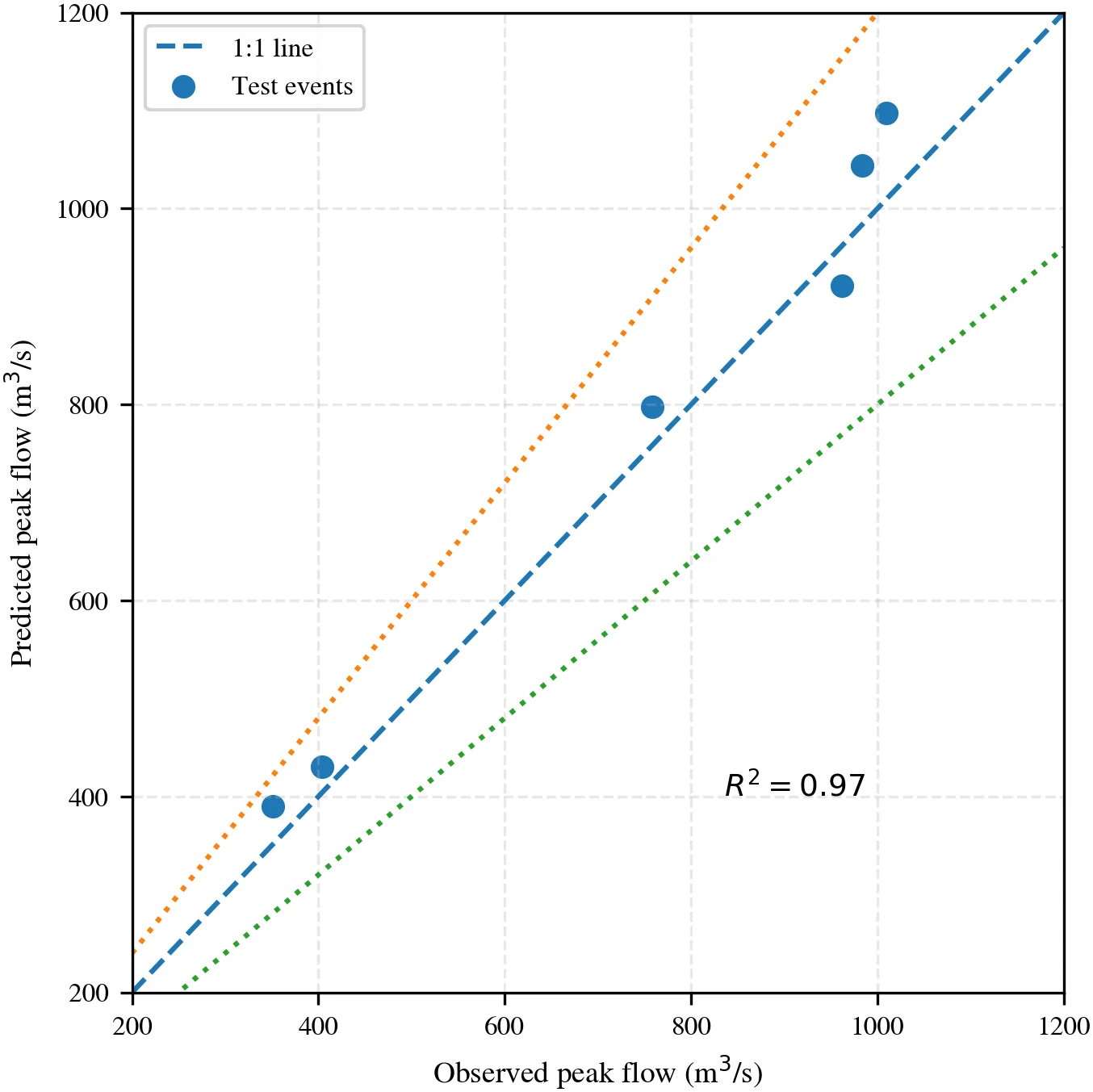
Observed versus predicted peak flows. Dashed: 1:1 reference; dotted: ±20% bounds. All points fall within the qualification bounds.

Fig 8 presents a scatter plot of observed versus predicted peak flows.

### Baseline model comparison

**Fig 9:**
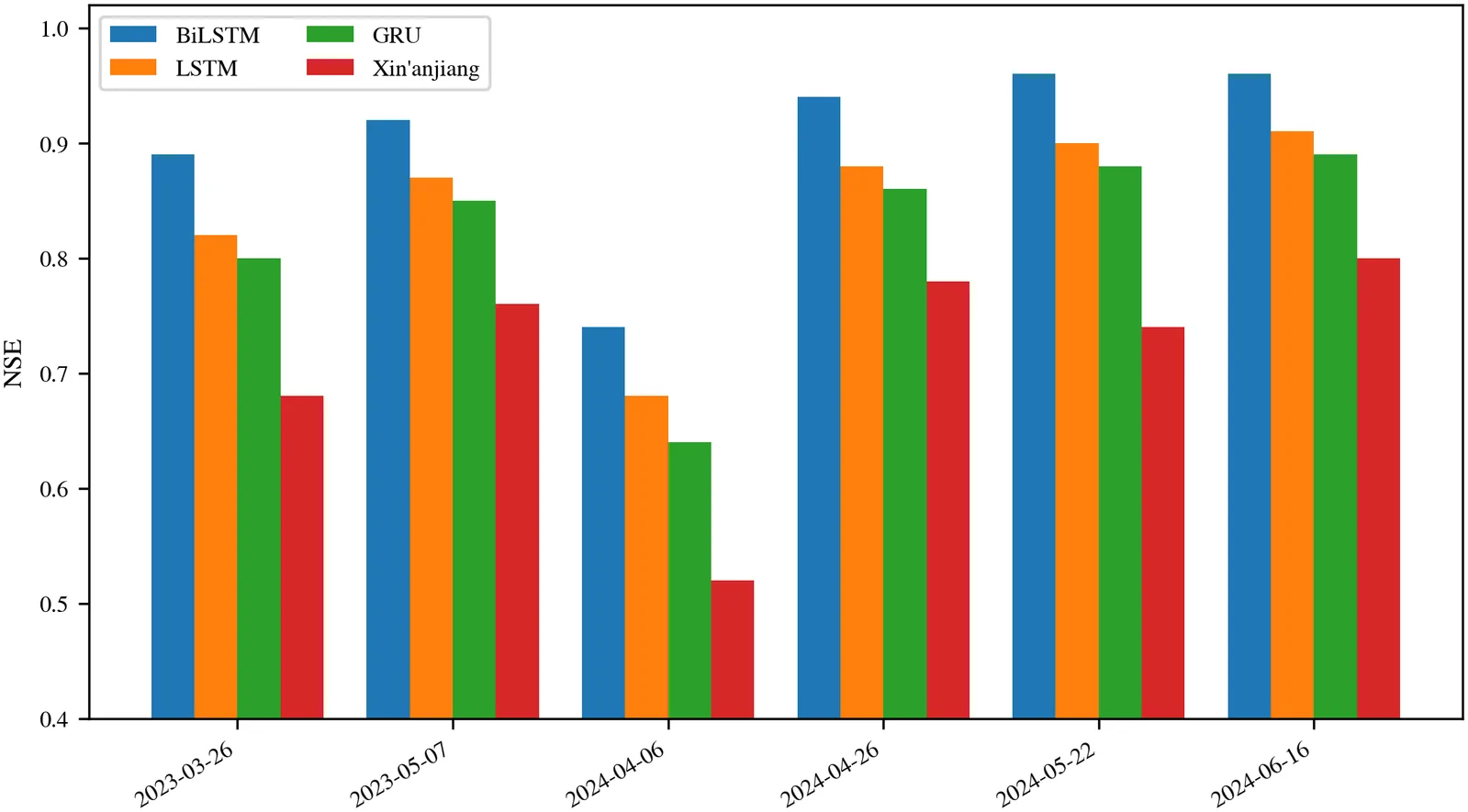
Per-event NSE comparison among BiLSTM, LSTM, GRU and Xin’anjiang.

Table 7 compares BiLSTM with the three baselines. BiLSTM achieves the highest mean NSE (0.90) and the lowest denormalised RMSE (11.2 m^3^/s). Only BiLSTM attains 100% qualification for both peak flow and timing. Fig 9 shows the corresponding event-level NSE values.

**Table 7:** Comparison of four models on six test events.

Model	Mean NSE	RMSE (m^3^/s)	|Peak err.| (%)	Peak flow qual.	Peak time qual.
**BiLSTM**	**0.90**	**11.2**	**6.98**	**100%**	**100%**
LSTM	0.84	16.8	10.5	83.3%	83.3%
GRU	0.82	18.4	12.1	83.3%	66.7%
Xin’anjiang	0.71	28.5	17.2	50.0%	50.0%

### Small-sample paired comparison

All six event-level NSE differences are positive for each baseline (Table 8). This directional consistency is not driven by a single event: deleting any one event leaves the mean improvement between 0.056 and 0.060 for LSTM, 0.078 and 0.084 for GRU, and 0.182 and 0.194 for Xin’anjiang. Nevertheless, *n* = 6 yields a wide and discrete inferential basis. The exact sign-test probability of 0.0156 should therefore be read as exploratory evidence of consistent direction, not as a precise estimate of generalisation to new basins or rare extreme floods.

**Table 8:** Event-level paired NSE differences and leave-one-event-out sensitivity.

Comparison	Positive pairs	Median ΔNSE	Range	Exact one-sided sign *p*	LOEO mean range
BiLSTM–LSTM	6/6	0.060	0.050–0.070	0.0156	0.056–0.060
BiLSTM–GRU	6/6	0.080	0.070–0.100	0.0156	0.078–0.084
BiLSTM–Xin’anjiang	6/6	0.185	0.160–0.220	0.0156	0.182–0.194

### SHAP feature analysis

**Fig 10:**
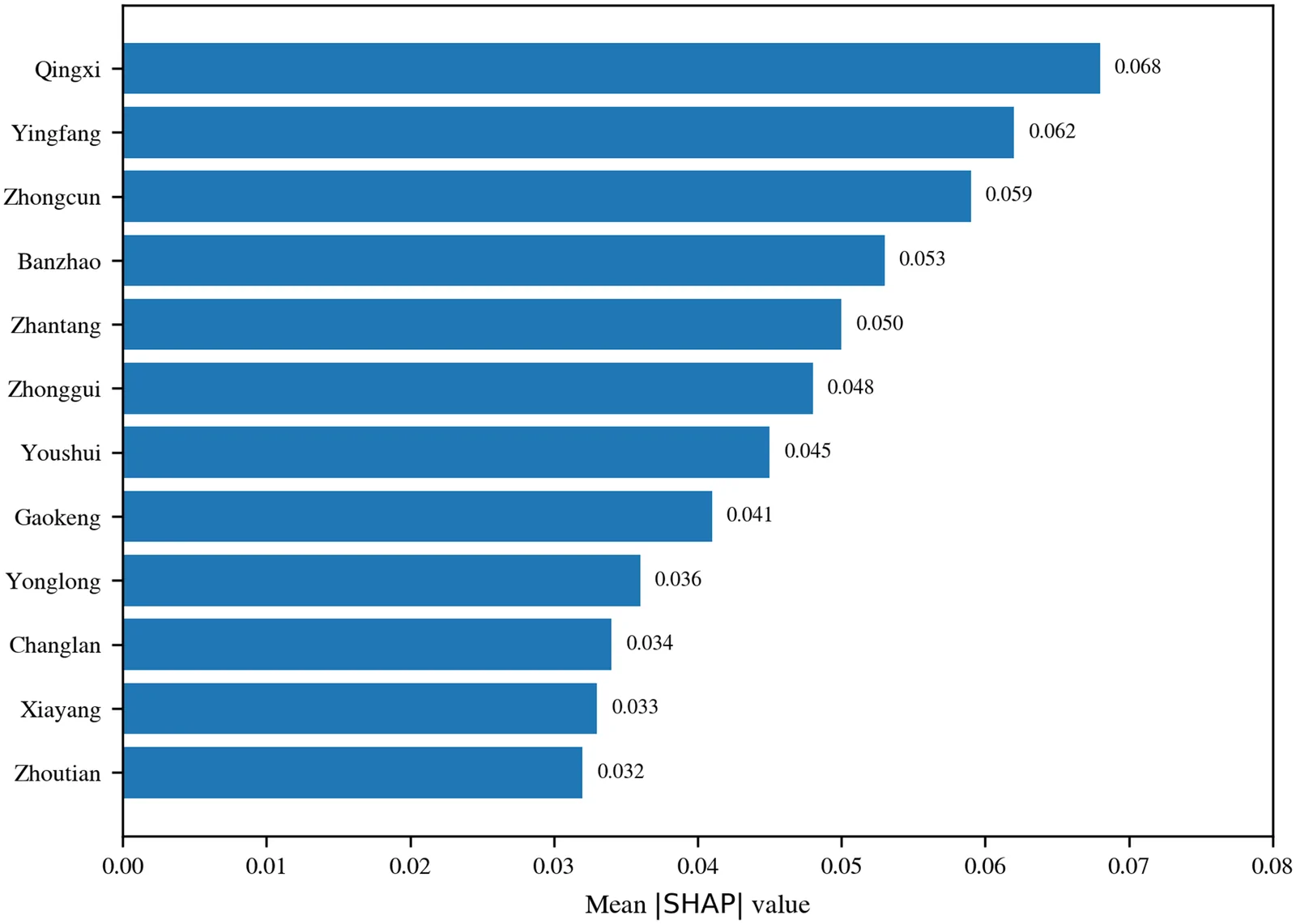
Mean absolute SHAP values per rain-gauge station. Upstream stations dominate.

Fig 10 shows station-level SHAP importance. Upstream stations Qingxi ($\overset{―}{\left|\phi \right|}=0.068$), Yingfang (0.062) and Zhongcun (0.059) rank highest, consistent with their proximity to the headwater area where steep terrain produces shorter concentration times.

**Fig 11:**
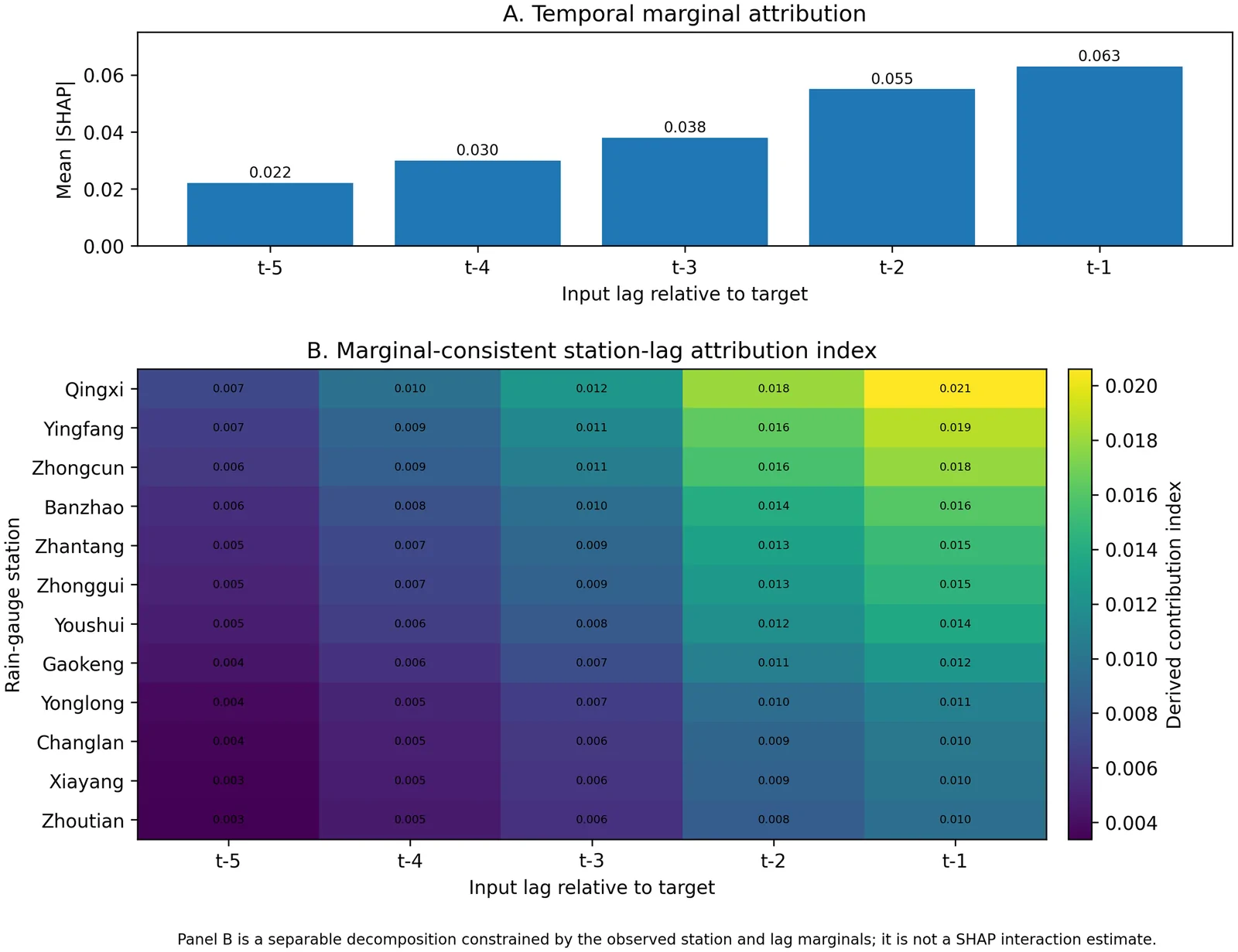
Temporal SHAP marginal and marginal-consistent station–lag attribution. A: Mean absolute SHAP by input lag. B: Derived station–lag attribution index from Eq 12. Panel B is a first-order diagnostic constrained by observed marginals and is not a SHAP interaction estimate.

Fig 11A shows that t−1 and t−2 dominate the temporal marginal. The marginal-consistent station–lag surface in Fig 11B localises the largest first-order indices at Qingxi, Yingfang and Zhongcun during these two lags. This is consistent with the short headwater-to-outlet concentration pathway described in Table 9. Because Eq 12 assumes separability, the heatmap cannot establish nonlinear interaction between a station and a lag; it identifies where the two verified marginals overlap most strongly.

**Table 9:** Underlying-surface characteristics and hydrological implications of the modelling domain.

Attribute	Available description	Implication for forecasting and transferability
Terrain	Hilly to mountainous; short steep slopes and narrow valleys	Short concentration time and strong sensitivity to upstream rainfall
Soil	Relatively shallow weathered soil in headwater areas	Rapid saturation and limited storage during intense storms
Land use	Forest-dominated uplands, cultivated valleys and rural settlements	Mixed infiltration response; less transferable to densely urbanised basins
Flood genesis	Mainly convective and monsoon storm-driven flash floods	Observed rainfall supports short leads; QPF is required at 3–6 h

### Spatio-temporal error analysis

**Fig 12:**
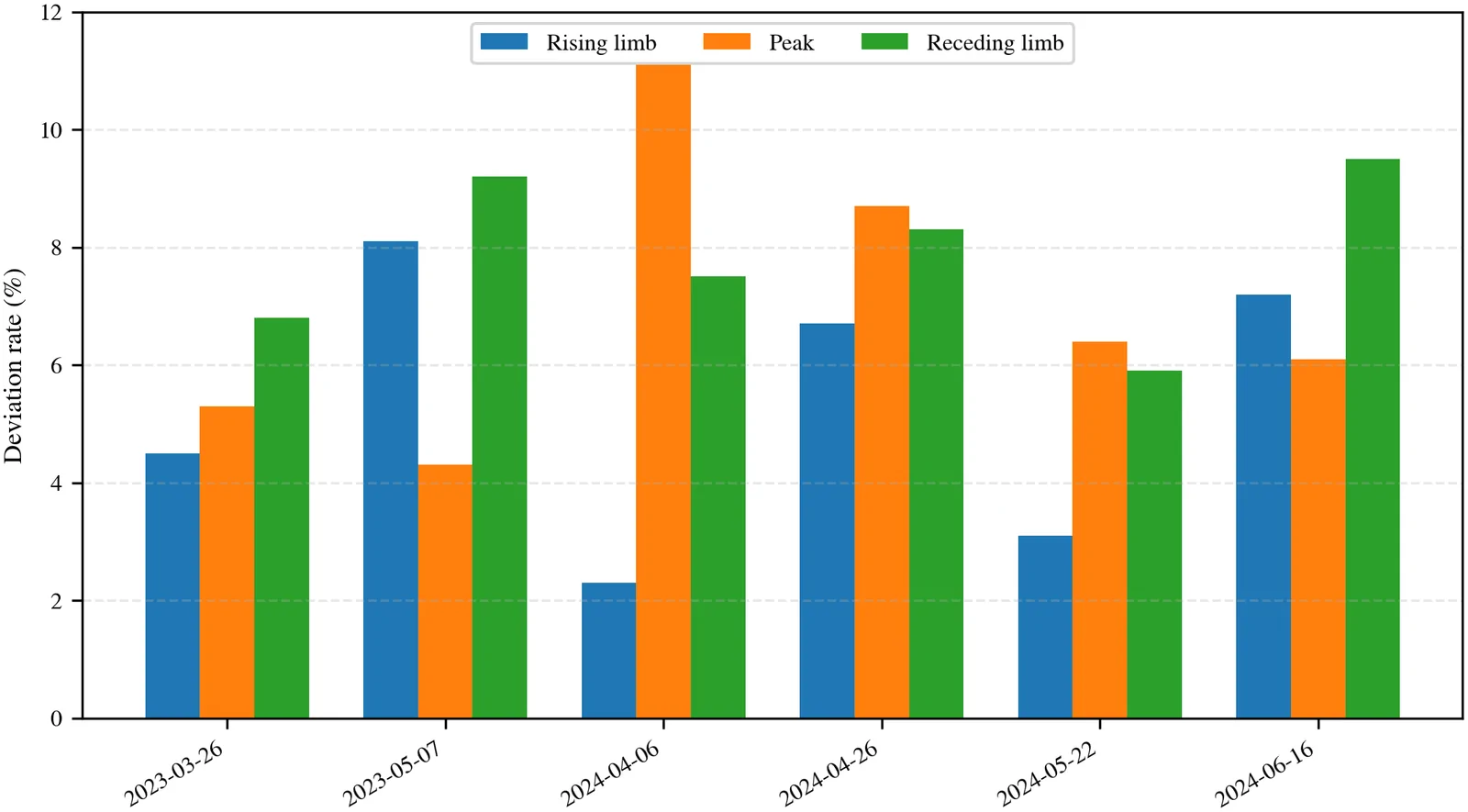
Deviation rates at different flood stages (rising, peak and receding) for each test event.

Fig 12 decomposes errors by flood phase. The rising-limb deviation is lowest (5.8%); peak averages 6.98% with no underestimation; the receding limb is highest (7.2%), especially for long-duration events (2024-06-16: 9.5%), because recession is governed by slow variables (soil moisture and groundwater) absent from the input features.

### Lead-time sensitivity

**Fig 13:**
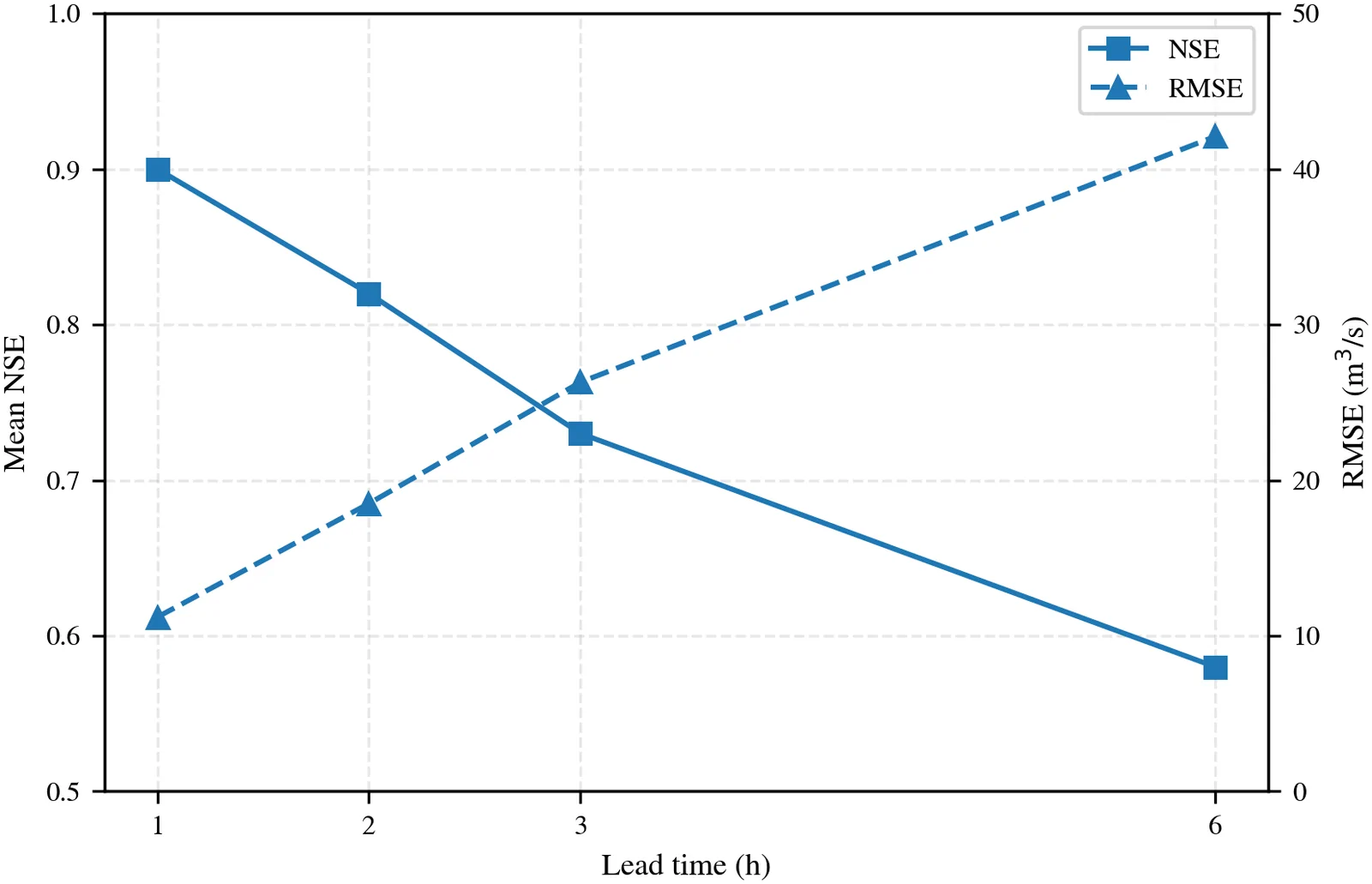
Lead-time-dependent forecast skill under an observed-only input structure. Skill declines rapidly beyond 2 h when no forecast rainfall forcing is supplied.

Fig 13 shows skill degradation with lead time: NSE decreases from 0.90 at 1 h to 0.82 at 2 h, 0.73 at 3 h and 0.58 at 6 h. The experiment keeps the observed-only input structure fixed, so the curve isolates how rapidly skill is lost when no future rainfall forcing is available. It should not be interpreted as a complete 3–6 h operational forecasting system. Table 10 specifies how the information structure must change with lead time.

**Table 10:** Required input information for different operational lead times.

Lead	Information available in the present experiment	Required operational extension
1 h	Recent observed rainfall at 12 stations	Rolling observation update; optional discharge/soil-moisture state
2–3 h	Same observed antecedent window	Radar extrapolation or short-range precipitation nowcast with uncertainty
6 h	Same observed antecedent window; inadequate for future forcing	Ensemble QPF rainfall, forecast-bias correction and probabilistic flow thresholds

## Early warning response mechanism

### The “631” mechanism

The “631” mechanism is a hierarchical, time-segmented emergency response system for flash-flood prevention in Jiangxi Province [27,28]. The “6” denotes a 6-hour provincial risk warning; “3” a 3-hour municipal notification; and “1” a 1-hour county-level response. The core “call-and-response” link comprises *wake-up* (ensuring warnings reach grassroots personnel via phone, broadcast and door-to-door contact) and *response* (immediate confirmation, inspection, evacuation and feedback).

**Fig 14:**
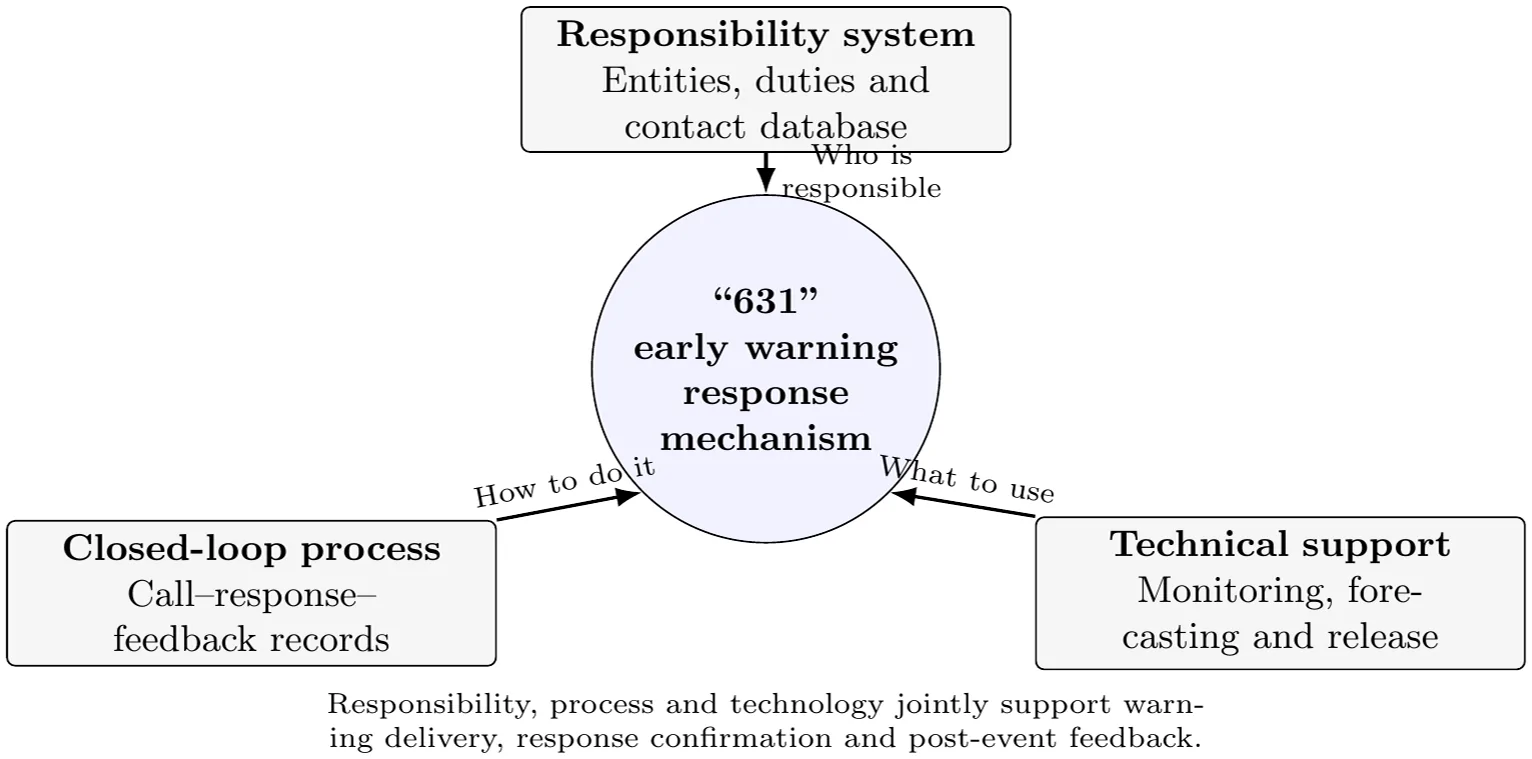
Composition and logical structure of the “631” early warning response mechanism.

Three supporting systems underpin the mechanism: (i) a five-level organisational hierarchy (province–county–township–village–household) with dynamically updated responsible-person databases; (ii) digital closed-loop management with mandatory call-back within 10 min of red alerts and multimedia feedback; and (iii) technical support from meteorological radars, rain gauges, hydrological stations and a unified command platform. Fig 14 illustrates the logical structure.

### Differentiation from other provincial mechanisms

While the “631” mechanism shares the general goal of hierarchical warning with the schemes in Zhejiang, Hunan and Gansu (Table 2), it differs in three key respects. First, it employs a *fixed time-segmented* trigger structure (6/3/1 h) rather than a purely dynamic or threshold-based approach, providing clear operational timelines for each administrative level. Second, it mandates *individual-level call-back confirmation*, creating an auditable chain of responsibility absent from grid- or map-based systems. Third, its closed-loop feedback cycle—with required photo/video evidence—enables post-event performance evaluation. However, Zhejiang’s digital-twin approach achieves faster automated response (<5 min vs. < 10 min), and Hunan’s multi-element map provides richer spatial risk context. These complementary strengths suggest that future upgrades should integrate the time-segmented structure with automated digital delivery and multi-element risk mapping.

### Integration of BiLSTM with the “631” framework

**Fig 15:**
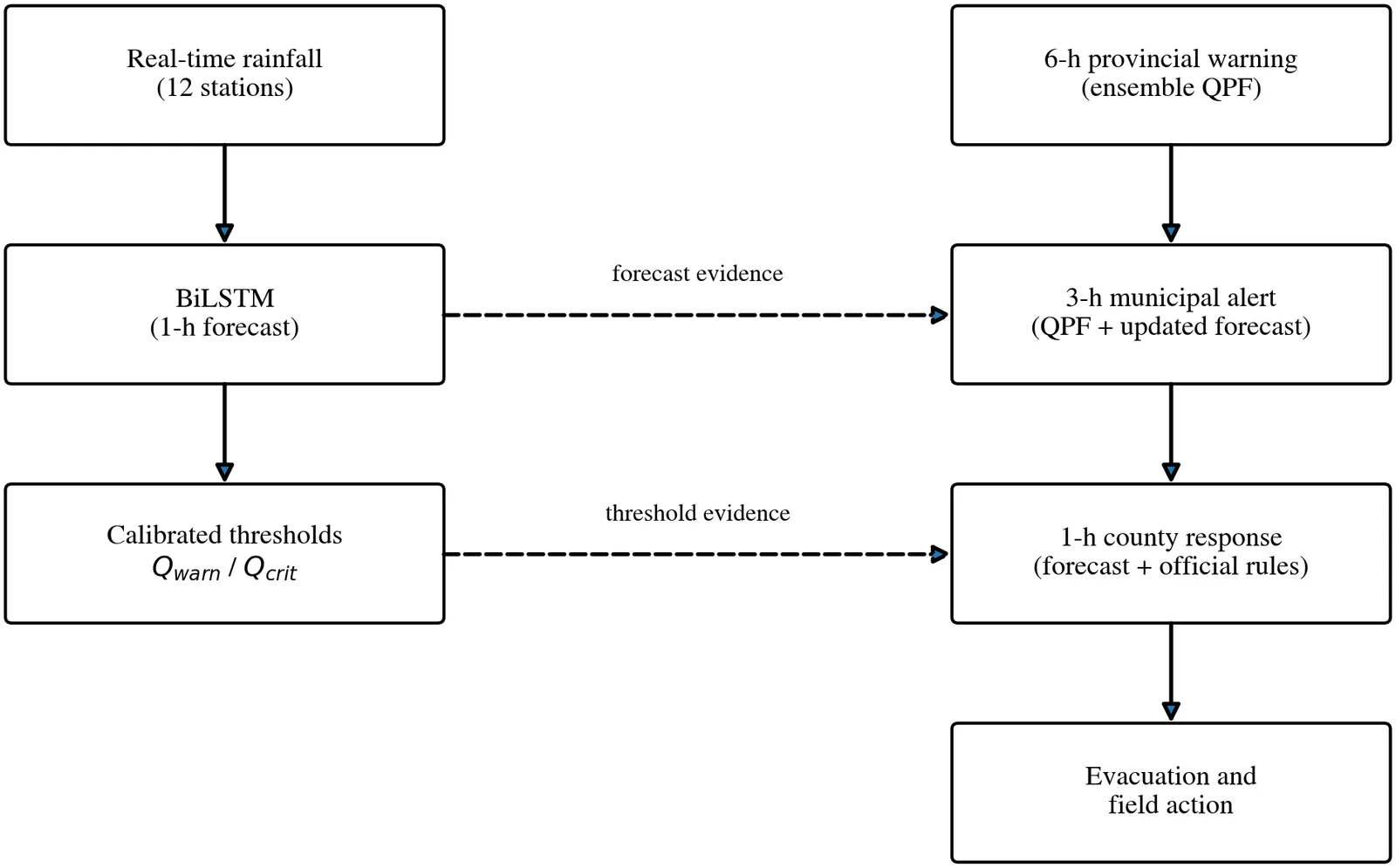
Integration of BiLSTM real-time forecasts with the “631” hierarchical trigger. BiLSTM outputs dynamically adjust the 3-h and 1-h thresholds.

A critical limitation of the original “631” mechanism is its reliance on *fixed rainfall thresholds* that do not account for antecedent moisture or spatial rainfall distribution. We propose an integration pathway (Fig 15) whereby BiLSTM forecasts dynamically adjust the trigger:

1. At the 6-h stage, provincial quantitative precipitation forecasts (QPF) trigger the initial warning. BiLSTM is initialised with the latest observations.
2. At the 3-h stage, BiLSTM produces rolling 1-h forecasts. If the predicted flow exceeds a locally calibrated warning-flow threshold *Q*_warn_, the municipal alert is elevated.
3. At the 1-h stage, if the forecast exceeds a locally calibrated critical-flow threshold *Q*_crit_, a county-level red alert is considered together with official stage, rainfall and field information. Thresholds must be calibrated from the station rating curve and approved emergency plan before operational use.

This design converts the “631” from a purely rainfall-threshold system into a hybrid forecast–threshold workflow. Its effect on lead time and false alarms must be verified prospectively before deployment.

### Case study: Fengcheng flash flood (2022)

**Fig 16:**
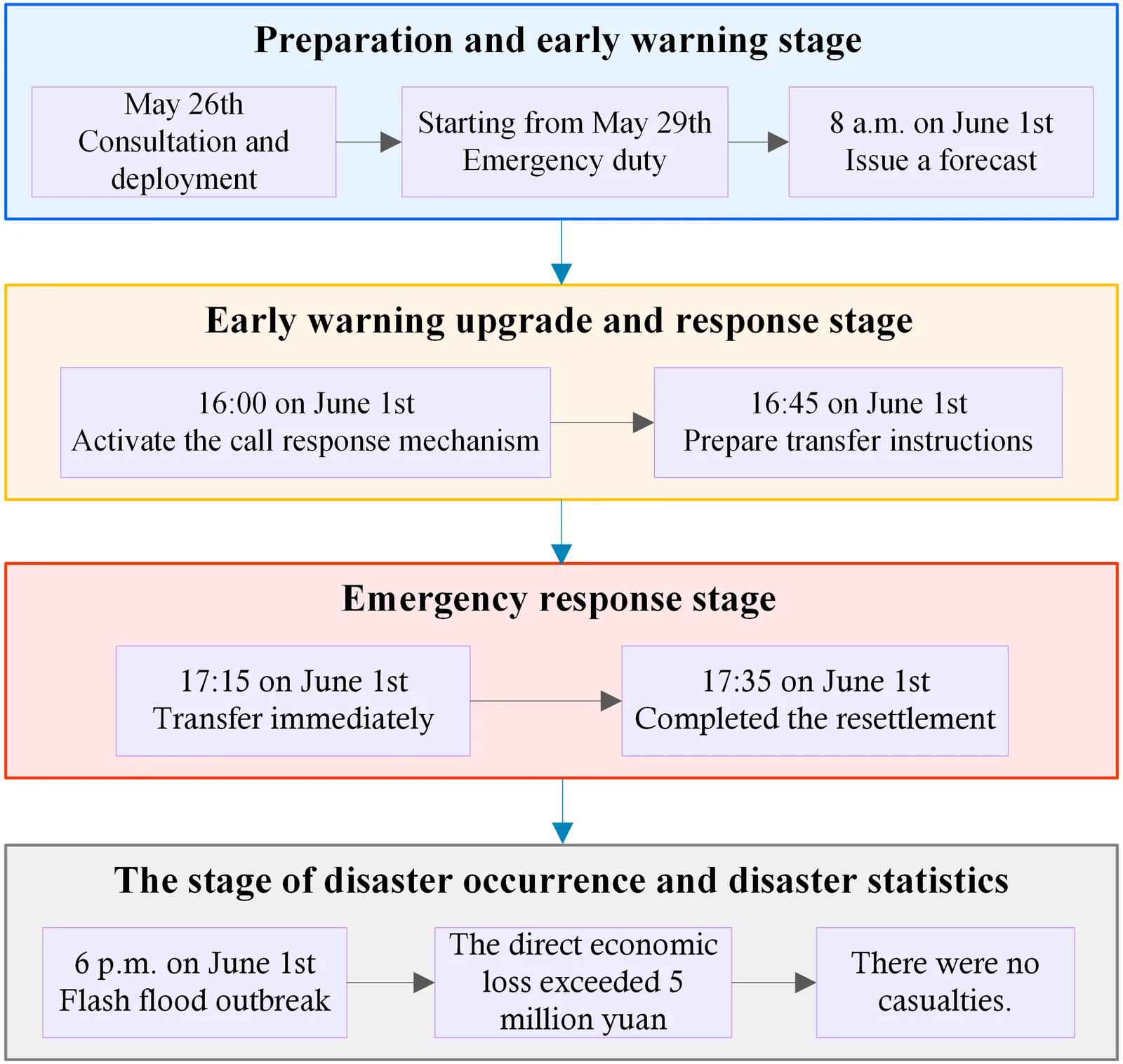
Key event timeline of the 2022 Fengcheng flash flood evacuation enabled by the “631” mechanism.

On 1 June 2022, Luoshi Town in Fengcheng City experienced a sudden heavy-rainfall event that provides an illustrative operational case for the “631” mechanism. Key timeline (Fig 16):

- 16:00 — Fengcheng Meteorological Bureau activated the 1-h risk response; the municipal flood office contacted Luoshi Town leaders via phone and WeChat.
- 16:45 — Xiaoxi Village rainfall reached 40 mm; the municipal office called again for evacuation preparation.
- 17:15 — Rainfall increased to 70 mm; the town government ordered immediate evacuation.
- 17:35 — All 62 residents (33 households) were evacuated to the resettlement site within 20 min.
- 18:00 — Rainfall reached 104.5 mm; severe flooding destroyed roads and houses; direct losses exceeded 5 million CNY. **Zero casualties.**

### Operational evidence and limitations

The Fengcheng chronology documents that repeated contact, local confirmation and early evacuation were completed before the most damaging stage of the event. It demonstrates the feasibility of the “call–response–feedback” chain, but a single case cannot isolate the causal effect of the mechanism or quantify a general reduction in casualties. A prospective evaluation should record warning issue time, receipt time, acknowledgement, evacuation start, false alarms and missed events across multiple counties.

Implementation still faces communication outages, limited accessibility for elderly residents, uneven grassroots staffing and incomplete inter-departmental data exchange. These constraints motivate a focused data-intelligence pathway rather than speculative automation: (i) integrate meteorological, hydrological and field observations with traceable timestamps; (ii) issue probabilistic, lead-specific forecasts whose uncertainty is visible to decision makers; and (iii) log warning delivery, acknowledgement and action so that forecast quality and response quality can be evaluated separately.

### Operational data-intelligence pathway

The revised pathway contains three auditable components. First, a common time-stamped data layer combines gauge rainfall, outlet flow, radar/QPF products and field reports. Second, a forecast layer produces lead-specific flow distributions rather than a single deterministic value; the 1-h product may rely primarily on observations, whereas 3–6 h products require precipitation forecasts. Third, a response layer records which warning rule was triggered, who received it, when it was acknowledged and which action followed. This separation allows forecast errors, communication failures and response delays to be diagnosed without attributing all outcomes to the prediction model.

## Discussion

### Model performance and limitations

Within the supplied Xiangshui event archive, BiLSTM consistently improves event-level NSE over LSTM, GRU and Xin’anjiang (Table 8). The result supports the usefulness of reversing the observed input window, but it does not establish superiority over attention-based or hybrid models that were not trained here. The SHAP station and lag marginals, together with the transparent station–lag index, align model attribution with the basin’s short, upstream-dominated concentration pathway without claiming causal or interaction effects.

Three limitations must be acknowledged. First, *data dependence*: only 20 events are available, with extreme events (24-h rainfall >200 mm) underrepresented. The paired leave-one-event-out sensitivity indicates that the six-event comparison is not driven by one test event, but extrapolation to beyond-design floods remains uncertain. Second, *physical interpretability*: despite SHAP providing input-level attribution, the model cannot explicitly represent processes such as soil-moisture dynamics or groundwater recharge. Future work should explore hybrid “data + physics” approaches, embedding process variables (soil moisture, evapotranspiration, terrain slope) in the input layer. Third, *receding-limb accuracy*: the 7.2% mean recession deviation (vs. 5.8% rising limb) reflects the absence of slow-process variables, motivating the inclusion of antecedent moisture indices.

Advanced architectures offer capabilities not tested here. Transformer/Informer and Pathformer models provide long-range or multi-scale attention [21,31]; two-dimensional hidden and DTTR models provide richer spatio-temporal or multimodal fusion [19,20]; and informed or hybrid models can embed hydrological constraints under limited data [18,32]. A fair next benchmark should use the same multi-basin split, lead-time-specific forcings, parameter-budget reporting and uncertainty evaluation. Until then, the present conclusion is limited to data efficiency and consistency against the three tested baselines.

### Mechanism effectiveness and integration value

The Fengcheng case illustrates how a time-segmented warning and mandatory call-back can create operational clarity and accountability. It does not, by itself, quantify population-level effectiveness. Comparative strengths in Table 2 should therefore be interpreted as differences in mechanism design rather than ranked performance.

The proposed integration addresses a critical disconnect: how model outputs can inform operational decisions without treating a model forecast as an automatic warning. Locally approved rainfall and water-level rules remain the formal basis for action; lead-specific flow forecasts can provide additional evidence and uncertainty information. For the 6-h stage, QPF rainfall should be assimilated to extend the input structure beyond observations. For the 3-h and 1-h stages, radar nowcasts, rolling rainfall and updated discharge forecasts can progressively refine the evidence available to duty officers.

### Generalisation and future directions

The findings are most directly relevant to humid, storm-driven mountainous basins with dense gauge coverage. Transfer to snowmelt-dominated, highly urbanised or arid catchments requires different process variables, forcing data and hydraulic representations. The next evaluation should therefore prioritise three testable extensions: multi-basin external validation, QPF-driven probabilistic forecasting at 3–6 h, and prospective logging of forecast issuance, warning receipt and response timing.

## Conclusion

The principal scientific insight is not that bidirectionality is universally superior, but that a compact recurrent model can provide useful short-lead information when the available evidence is restricted to a small archive of storm events. Across the six held-out events, every NSE difference favoured BiLSTM over the three tested baselines; however, the discrete small-sample analysis and the absence of external-basin validation prevent a population-level superiority claim.

The attribution results connect model behaviour to basin response at two verified marginal levels. Upstream stations carry the largest station importance, and the most recent two lags carry the largest temporal importance. Their overlap in the marginal-consistent station–lag diagnostic is hydrologically plausible for a steep, shallow-soil, storm-driven basin, but it remains a first-order decomposition rather than evidence of nonlinear SHAP interaction or causality.

Operationally, the lead-time experiment defines a boundary condition for integrating forecasts with the “631” mechanism. Observation-driven BiLSTM forecasts are most defensible at the 1-h stage. The 3-h and 6-h stages require radar nowcasts or ensemble QPF, bias correction and uncertainty-aware thresholds; extending the same observed-only input window is insufficient. A future multi-basin study should therefore combine raw-data and code release, genuine station–lag interaction analysis, advanced attention and hybrid baselines, and prospective logging of both forecast and warning-response outcomes.

## Supporting information

S1 Data The supporting-information upload contains the machine-readable numerical values underlying the manuscript tables and programmatic figures, including event-level BiLSTM metrics, event-level baseline NSE values, station GRA and SHAP marginals, temporal SHAP marginals, the derived station–lag index, flood-phase deviations, lead-time sensitivity and hyperparameter results. (ZIP)

S1 Code The supporting-information upload contains a transparent, event-safe reimplementation of data validation, model fitting, evaluation and SHAP export. It includes dependency specifications, deterministic seed controls, tests and a synthetic-data smoke example. Because the original hourly records, trained weights and original training scripts were not available in the archived materials, this reimplementation was not used to generate the numerical results reported in this article.
(ZIP)
